# The Mms22-Rtt107 axis dampens the DNA damage checkpoint by reducing the stability of the Rad9 checkpoint mediator

**DOI:** 10.21203/rs.3.rs-4417144/v1

**Published:** 2024-05-24

**Authors:** Xiaolan Zhao, Bingbing Wan, Danying Guan, Shibai Li, Tzippora Chwat-Edelstein

**Affiliations:** Memorial Sloan Kettering Cancer Center; Shanghai Jiao Tong University; Memorial Sloan Kettering Cancer Center; Memorial Sloan Kettering Cancer Center; Memorial Sloan Kettering Cancer Center

**Keywords:** DNA damage checkpoint, checkpoint dampening, Rtt107, Mms22, Slx4

## Abstract

The DNA damage checkpoint is a highly conserved signaling pathway induced by genotoxin exposure or endogenous genome stress. It alters many cellular processes such as arresting the cell cycle progression and increasing DNA repair capacities. However, cells can downregulate the checkpoint after prolonged stress exposure to allow continued growth and alternative repair. Strategies that can dampen the DNA damage checkpoint are not well understood. Here, we report that budding yeast employs a pathway composed of the scaffold protein Rtt107, its binding partner Mms22, and an Mms22-associated ubiquitin ligase complex to downregulate the DNA damage checkpoint. Mechanistically, this pathway promotes the proteasomal degradation of a key checkpoint factor, Rad9. Furthermore, Rtt107 binding to Mms22 helps to enrich the ubiquitin ligase complex on chromatin and target the chromatin-bound form of Rad9. Finally, we provide evidence that the Rtt107-Mms22 axis operates in parallel with the Rtt107-Slx4 axis, which displaces Rad9 from chromatin. We thus propose that Rtt107 enables a bifurcated “anti-Rad9” strategy to optimally downregulate the DNA damage checkpoint.

## INTRODUCTION

The highly conserved DNA damage checkpoint (DDC) is a critical component of the genome stress response^1–3^. When cells suffer from increased burdens of genome lesions caused by genotoxins or endogenous sources, the DNA damage sensor proteins can recruit apical DDC kinases to DNA lesion sites leading to their subsequent activation^4, 5^. DDC mediator proteins can then amplify the genome stress signals by recruiting downstream transducer kinases, which can be phosphorylated and activated by the apical DDC kinases^6–8^. The activated transducer kinases can diffuse to various cellular compartments and phosphorylate hundreds of substrates^9^. Such a large-scale phosphoproteomic transformation can induce a myriad of cellular changes. These include cell cycle arrest to provide more time for DNA repair, increased ability to repair, and transcriptional alterations, among many other consequences^3, 10^. While DDC-induced changes are beneficial for cells to cope with stress temporarily, long-term survival also depends on the ability to dampen the checkpoint once stress is dealt with or when stress becomes persistent^11–13^. Checkpoint dampening can permit cell cycle resumption and thus the chance of continued growth, as well as access to DNA repair mechanisms operating in different cell cycle phases, among other benefits^11–13^. However, the various strategies employed to dampen DDC remain to be discovered.

DDC dampening has thus far been mostly examined in the model organism budding yeast. Initial studies have focused on protein phosphatases that can reverse some of the phosphorylation changes elicited by the DDC kinases^11^. However, phosphatases alone are insufficient for DDC dampening, and cells also need to reduce the continuous emission of DDC signals generated through the chains of the DNA damage sensors, apical DDC kinases, checkpoint mediators, and transducer kinases. Examples of phosphatase-independent strategies to downregulate DDC include displacing checkpoint factors from chromatin^14–18^. Whether and how additional pathways can reduce DDC signaling remain to be elucidated.

Here we report a strategy of DDC dampening mediated by proteasomal degradation of a key checkpoint mediator protein Rad9 in yeast. Rad9 associates with DNA lesion sites in response to genotoxin treatment through binding to nucleosomes containing γH2A that demarcate damage domains and methylated H3K79^19, 20^. Rad9 can then recruit the transducer kinase Rad53 in preparation for Rad53 phosphorylation and subsequent activation by the main apical DDC kinase, Mec1^21, 22^. As Rad53 is responsible for a large majority of DDC-induced phosphorylation events in yeast, regulating its upstream enabler, Rad9, can be a key step to downregulate DDC.

Indeed, a scaffolding complex composed of the Rtt107 and Slx4 proteins was shown to compete with Rad9 for binding to nucleosomes, thus reducing Rad9’s association with DNA lesion sites^14, 23^. Interestingly, Rtt107 can additionally bind to another scaffold protein Mms22^24–26^. Mms22 was shown to serve as a substrate adaptor of the cullin ubiquitin E3 complex, which also contains the E3 subunit Rtt101 and the Mms1 subunit (Rtt101^Mms1^ E3)^27^. While the Mms22-Rtt101^Mms1^ E3 complex is known to protect genome stability and support cell growth when facing genotoxins, its functional mechanisms are not fully understood^28^. We show here that Rtt107 binding to Mms22 as well as the Rtt101^Mms1^ E3 are required for Rad9 degradation. We also demonstrate that Mms22 and Slx4 act in parallel to achieve a more effective “anti-Rad9” outcome during DDC recovery. As such, Rtt107 enables a two-pronged strategy to downregulate Rad9-mediated DDC via collaborating with its two binding partners.

## RESULTS

### Rtt107 association with Mms22 prevents genotoxin sensitivity and prolonged checkpoint

**Figure 1:**
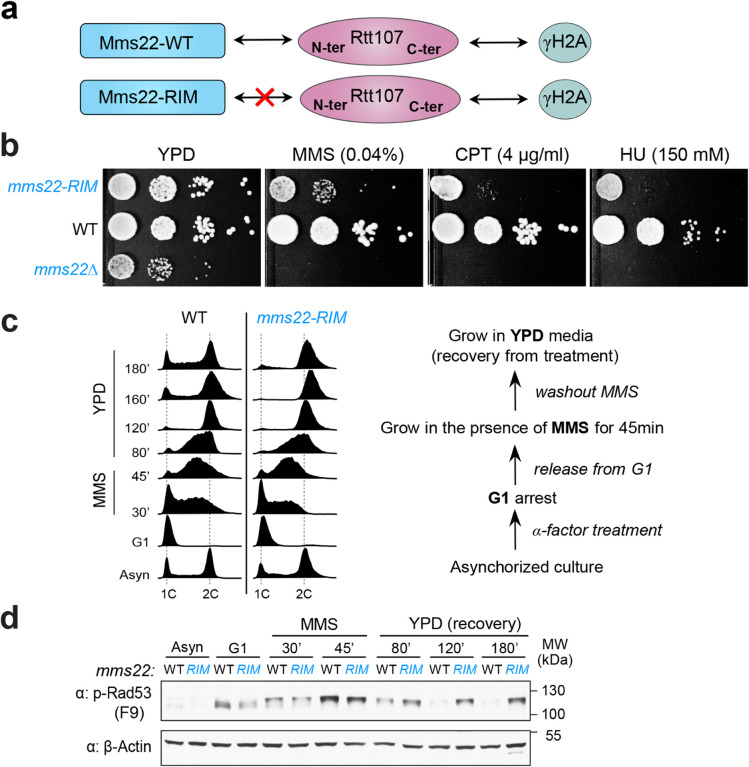
Disrupting Mms22 binding to Rtt107 leads to genotoxin sensitivity and persistent DNA damage checkpoint. **a** Schematic showing that Mms22 uses its RIM sequence to bind to the N-terminal (N-ter) tetra-BRCT domain of the scaffold Rtt107 protein, while gH2A binds to the C-terminal (C-ter) domain of Rtt107^29, 36^. The *mms22*^*RIM*^ mutant specifically disrupts Mms22 binding to Rtt107^29^. **b** *mms22*^*RIM*^ and *mms22Δ* cells exhibited sensitivity to three genotoxins. Cells were spotted in 10-fold serial dilutions. **c** *mms22*^*RIM*^ cells are defective in exiting the G2/M phase compared with wild-type cells after transient exposure to MMS. The experimental scheme is depicted on the right. Represented FACS profiles are shown on the left. **d** The active form of Rad53 persists in *mms22*^*RIM*^ but not in wild-type cells after transient exposure to MMS. Samples were taken in the same experiments as those in panel **c**. The active form of Rad53 was detected by the F9 antibody. β-Actin served as the loading control.

Both Rtt107 and Mms22 are important for genome stability; however, the biological functions of their interaction have been unclear. To address this question, we examined a separation-of-function allele of Mms22 that specifically disrupts the Rtt107 binding without affecting other known interactions^29^. Given that Rtt107 and Mms22 each have multiple binding partners, this allele provides a valuable reagent to define the biological functions of their interaction^25, 28, 30^. High-resolution structure has revealed that Rtt107 binds to the N-terminal Rtt107-interaction-motif (RIM) of Mms22^29^. Further, alanine substitution of two residues (D13 and Y33) within Mms22’s RIM (*mms22*^*RIM*^) disrupts the Mms22 and Rtt107 interaction (Fig. 1a)^29^. *mms22*^*RIM*^ supports the wild-type level of protein expression and interactions with other binding partners, such as the Mms1 protein that associates with the C-terminal region of Mms22 (Supplementary Fig. 1a)^25, 29^. We con rmed here that *mms22*^*RIM*^ led to sensitivity to the DNA methylation agent MMS (methyl methanesulfonate) and further showed that it also caused sensitivity to the Top1 trapping compound CPT (camptothecin) and the replication fork blocking agent HU (hydroxyurea) (Fig. 1b). Compared with *mms22*^*RIM*^, *mms22Δ* led to greater sensitivity to MMS, CPT, and HU, as well as slower growth (Fig. 1b). This agrees with the notion that *mms22*^*RIM*^ is a separation-of-function allele affecting the Mms22-Rtt107 binding without interfering with other roles of Mms22.

Sensitivity to several genotoxins raised the possibility that *mms22*^*RIM*^ may affect common processes during different genotoxin responses such as the DNA damage checkpoint. To test this notion and determine the underlying causes of the genotoxin sensitivity exhibited by *mms22*^*RIM*^ cells, we focused on MMS treatment since DDC has been well examined in this condition. Specifically, we examined how synchronized cells moved through the cell cycle in a regimen that allows monitoring of both the initial response to MMS and the recovery. Briefly, cells synchronized in G1 were released to the cell cycle in the presence of MMS for 45 minutes, and then allowed to recover in MMS-free media (Fig. 1c, right). As expected, wild-type cells progressed slowly through S phase and were able to enter the second cell cycle after MMS washout (Fig. 1c). We also examined Rad53 activation, a marker for DDC signaling. The F9 antibody is widely used to detect Rad53 activation as it specifically recognizes the phosphorylated but not unmodified form of Rad53, although the antibody also binds to a nonspecific band only in G1 cells^31^. As previously reported, we observed that in wild-type cells, Rad53 activation was induced in S phase when cells were treated with MMS (30–45 min) and gradually diminished during the recovery phase (Fig. 1d and Supplementary Fig. 1b).

In contrast to wild-type cells, *mms22*^*RIM*^ cells delayed the re-entry into the second G1 phase after MMS washout, though showed no obvious defects during S phase progression in the presence of MMS (Fig. 1c). Concomitantly, Rad53 phosphorylation induced by MMS failed to decline during the recovery phase (Fig. 1d and Supplementary Fig. 1b). The results from these two assays are consistent with each other and suggest that *mms22*^*RIM*^ cells exhibit persistent DDC during the recovery phase, which can delay entry to the next cell cycle.

#### Genotoxin sensitivity associated with the loss of the Mms22-Rtt107 interaction is rescued by reducing the DNA damage checkpoint

**Figure 2:**
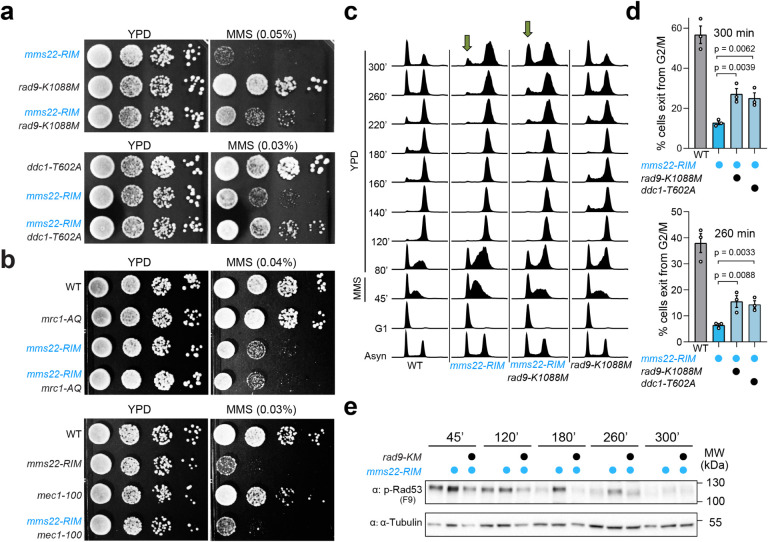
Three related phenotypes of *mms22*^*RIM*^ cells are rescued by mild mutant alleles of the DNA damage checkpoint proteins. **a** The MMS sensitivity of *mms22*^*RIM*^ cells is suppressed by point mutations in the DNA damage checkpoint protein Rad9 or Ddc1. Experiments were done as in Fig. 1b. **b** The MMS sensitivity of *mms22*^*RIM*^ cells is not affected by mutations reducing the DNA replication checkpoint, including mrc1-AQ and mec1–100. Experiments were done as in Fig. 1b. **c** Defects in exiting the G2/M phase seen in *mms22*^*RIM*^ cells are improved by mutating Rad9. FACS profiles of indicated strains are shown. Arrows highlight different amounts of cells that have exited the first G2/M phase and entered the second G1 phase at 300 min for two of the examined strains. Experiments were done as in Fig. 1c. **d** Percentages of cells that have exited the G2/M phase and entered the next G1 phase. The calculation was based on three biological duplicates for the indicated time points. Averages and Standard Error of the Means (SEMs) are indicated; statistical analysis was performed by one-tailed unpaired Student’s *t-*test. **e** Persistent Rad53 activation in *mms22*^*RIM*^ cells is suppressed by *rad9-K1088M*. Experiments were done as in Fig. 1d, and α-Tubulin served as the loading control.

Persistent checkpoint can be caused by delayed genome replication and repair or by reduced ability to dampen the checkpoint itself. The two scenarios differ in how cells would respond to reduced levels of DDC. In the first scenario, cell viability would suffer when checkpoint function is weakened, because cells rely on optimal checkpoint to complete DNA replication and repair. In contrast, cells in the second scenario could show better survival upon checkpoint weakening. Based on this rationale, we tested how *mms22*^*RIM*^ cells responded to reduced DDC levels conferred by mild alleles of the checkpoint mediator protein Rad9 or Ddc1. The two mutants used, namely *rad9-K1088M* and *ddc1-T602A*, contain single point mutations that reduce Rad9 binding to γH2A and Ddc1 binding to another checkpoint factor Dpb11^20, 32^. These studies have shown that *rad9-K1088M* and *ddc1-T602A* mildly reduce DDC. Significantly, we found that either allele conferred strong rescue of the MMS sensitivity of *mms22*^*RIM*^ cells (Fig. 2a).

While the DNA damage checkpoint can operate throughout the cell cycle, cells also employ the DNA replication checkpoint (DRC) during S phase^3^. We next examined whether mild hypomorphic DRC mutants could affect the MMS sensitivity of *mms22*^*RIM*^ cells. We tested two well-characterized alleles affecting either the Mrc1 mediator protein of the DRC pathway, namely *mrc1-AQ*, or *mec1–100* that is specifically defective in DRC^33, 34^. As shown in Fig. 2b, neither allele affected the MMS sensitivity of *mms22*^*RIM*^ cells. Together, these data suggest that defects of *mms22*^*RIM*^ cells can be rescued by reducing DDC, but not DRC, functions. Collectively, the genetic findings raise the possibility that Mms22 binding to Rtt107 contributes to the downregulation of DDC but not DRC, and that this role can be partly responsible for the genotoxin sensitivity caused by the loss of the Mms22-Rtt107 interaction.

#### Persistent checkpoint in mms22-RIM cells is rescued by rad9-K1088M and ddc1-T602A

To further test the above hypothesis, we examined whether the suppression of MMS sensitivity of *mms22*^*RIM*^ cells by the DDC mutants is associated with a correction of the persistent checkpoint seen in *mms22*^*RIM*^ cells. We used a similar experimental scheme as depicted in Fig. 1c to monitor cell cycle progression and Rad53 activation, except a longer period of recovery was examined. Similar to observations described above (Fig. 1c), while wild-type cells were able to enter the next cell cycle after recovery from MMS treatment, *mms22*^*RIM*^ delayed the entry (Fig. 2c and Supplementary Fig. 2a). This delay was quantified for two late time points (260 and 300 min; Fig. 2d). Significantly, this delay was improved by either *rad9-K1088M* or *ddc1-T602A* (Fig. 2c and Supplementary Fig. 2a). Compared with *mms22*^*RIM*^, more cells in the *mms22*^*RIM*^ *rad9-K1088M* or *mms22*^*RIM*^ *ddc1-T602A* double mutants exited G2/M and entered the next G1 phase between 200 to 300 minutes, with the most prominent differences seen at 260 and 300 minutes (Fig. 2d). *rad9-K1088M* and *ddc1-T602A* behaved similarly to wild-type cells, reflecting redundancy among DDC mediator functions (Figs. 2c and 2d, Supplementary Fig. 2a). Importantly, *rad9-K1088M* or *ddc1-T602A* also reduced the persistent Rad53 activation seen in *mms22*^*RIM*^ cells (Fig. 2e and Supplementary Fig. 2b). Collectively, the suppression of prolonged Rad53 activation and cell cycle arrest seen in *mms22*^*RIM*^ by *rad9-K1088M* or *ddc1-T602A* provides further evidence that Mms22 binding to Rtt107 plays a role in dampening the DNA damage checkpoint.

### Mms22 and Slx4 act in parallel to dampen the DNA damage checkpoint

Rtt107 has an established role in DDC dampening through pairing with Slx4^14, 23^. Rtt107 employs the same surface to engage with short motifs within Slx4 or Mms22, engendering mutually exclusive pairwise interactions^29^. A separation-of-function allele, *slx4*^*TTS*^ (T423, T424, and S567A), has been constructed that specifically abolishes the Slx4-Rtt107 interaction without affecting protein level or binding to other known partners, such as the Slx1 nuclease^29^. We thus asked how the Mms22-Rtt107 mediated effect on DDC recovery is functionally related to that mediated by Slx4-Rtt107.

**Figure 3:**
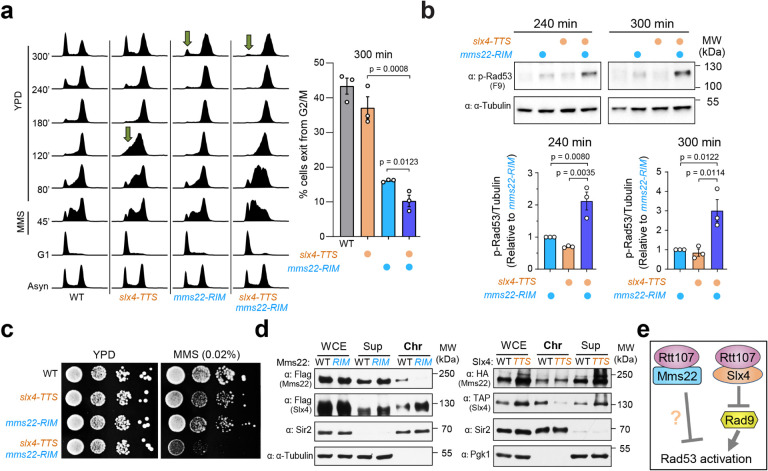
The Mms22-Rtt107 axis acts in parallel to the Slx4-Rtt107 axis in checkpoint dampening. **a** *mms22*^*RIM*^ and *slx4*^*TTS*^ are additive in delaying the exit from the G2/M phase after transient exposure to MMS. Experiments were done as in Fig. 1c. Left: FACS profiles of indicated strains, with arrows highlighting different amounts of G1 cells in the mutants toward the end of the time course. Right: percentages of cells that have exited the G2/M phase at the indicated time point were calculated based on three biological duplicates. Averages and SEMs are indicated; statistical analysis was performed by one-tailed unpaired Student’s *t*-test. **b** *mms22*^*RIM*^ and *slx4*^*TTS*^ show additive effect for increasing the levels of active Rad53. Experiments were done as in Fig. 1d. Top: representative immunoblotting results detecting Rad53 phosphorylation at indicated time points. Tubulin served as the loading control. Bottom: relative levels of phosphorylated Rad53 calculated based on three biological duplicates. Averages and SEMs are indicated; statistical analysis was determined by one-tailed unpaired Student’s *t*-test. **c** *mms22*^*RIM*^ and *slx4*^*RIM*^ are additive in causing MMS sensitivity. Experiments were done as in Fig. 1b. **d** The effects of *mms22*^*RIM*^ and *slx4*^*RIM*^ on the chromatin association of Mms22 and Slx4. Sir2 and Tubulin were used to mark chromatin-bound and non-chromatin-bound fractions, respectively. Left: cells contained Flag-tagged Mms22 and Slx4. Right: cells contained HA tagged Mms22 and TAP-tagged Slx4. **e** A model to summarize the Mms22-Rtt107 and Slx4-Rtt107 pathways in checkpoint dampening as suggested by our data presented in Figs. 1–3.

Applying the experimental scheme described in Fig. 2A, we found that *slx4*^*TTS*^ led to a more pronounced delay in late S phase compared with *mms22*^*RIM*^ (80 and 120 min; Fig. 3a). In contrast, *slx4*^*TTS*^ showed a milder delay in re-entry into the next cell cycle compared with *mms22*^*RIM*^ (240 and 300 min; Fig. 3a). These observations raised the possibility that the two Rtt107 interactors may differentially affect DDC at different stages of the cell cycle.

Significantly, the *mms22*^*RIM*^ *slx4*^*TTS*^ double mutant exhibited more severe defects in exiting the G2/M arrest in the first cell cycle than either single mutant (Fig. 3a). At the end of the time course (300 min), the double mutant showed significantly fewer cells exited the G2/M phase than either single mutant (Fig. 3a). A similar additive effect was seen when assaying for the active form of Rad53. The *mms22*^*RIM*^*slx4*^*TTS*^ double mutant showed higher levels of active Rad53 than either single mutant at the two last time points of the time course (240 and 300 min; Fig. 3b). Results from cell cycle analysis and active Rad53 forms can be best explained by that Mms22-Rtt107 and Slx4-Rtt107 complexes acting in different pathways to downregulate the DNA damage checkpoint.

### Additional evidence supports the independence of Mms22- and Slx4- mediated functions

Further supporting the functional independence of Mms22 and Slx4, we found that the double mutant of *mms22*^*RIM*^*slx4*^*TTS*^ led to stronger MMS sensitivity than either single mutant (Fig. 3c). The additive effect of *mms22*^*RIM*^ and *slx4*^*TTS*^ was also seen when assaying the stability of the repetitive ribosomal DNA (rDNA) locus and of a non-rDNA locus during growth, suggesting that their independence is a general feature (Supplementary Fig. 3). We showed that each single mutant caused a 4 to 5-fold increase in marker loss at rDNA, while the *mms22*^*RIM*^*slx4*^*TTS*^ double mutant led to a 10-fold increase (Supplementary Fig. 3a). Similarly, in the gross chromosome rearrangement (GCR) assay that assesses the stability of a non-rDNA locus, we detected a 59-fold increase of marker loss in the *mms22*^*RIM*^*slx4*^*TTS*^ double mutant and a 16 to 20-fold increase in the corresponding single mutants (Supplementary Fig. 3b). These genetic data are in line with the results from two cell cycle checkpoint assays described above as well as previous biochemical findings that Mms22 and Slx4 partner with Rtt107 in a mutually exclusive manner^29^. Together, they strongly suggest that Mms22 and Slx4 represent parallel pathways in regulating both the DNA damage checkpoint and genomic stability.

While our data support the functional independence of Mms22 and Slx4, the two proteins have the potential to indirectly affect each other due to sharing a common partner, Rtt107, that facilitates the chromatin recruitment of both^29^. To examine this possibility, we asked if Mms22 and Slx4 affect each other’s chromatin association. We queried how the loss of the Slx4-Rtt107 interaction could affect Mms22 chromatin association and vice versa. We confirmed the reported findings that *mms22*^*RIM*^ and *slx4*^*TTS*^ each reduced its own chromatin association (Fig. 3d). In contrast, *mms22*^*RIM*^ led to an increase in Slx4 level on chromatin while *slx4*^*TTS*^ showed no effect on the chromatin association of Mms22 (Fig. 3d and Supplementary Fig. 3c). These results suggest that the checkpoint dampening defects seen for *mms22*^*RIM*^ cells are not due to an indirect effect of reducing Slx4 chromatin association, and vice versa. This notion is consistent with data describing the additive effect of *mms22*^*RIM*^ and *slx4*^*TTS*^ shown above (Figs. 3a–3c); together they suggest that Mms22 and Slx4 can work in parallel pathways with each collaborating with Rtt107 (Fig. 3e).

### The Mms22-Rtt107 interaction and the Rtt101 E3 promote Rad9 degradation

**Figure 4:**
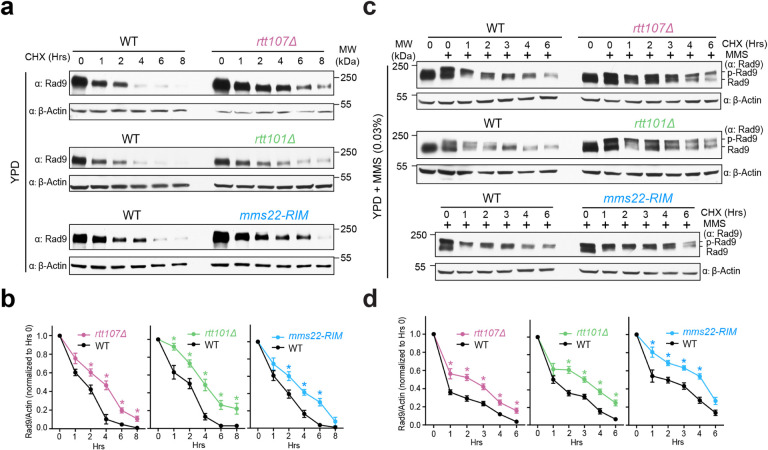
Mms22, Rtt107, and the Rtt101^Mms1^ E3 contribute to Rad9 instability. **a** Rad9 protein stability examined in the presence of cycloheximide (CHX) that blocks new protein synthesis. Both wild-type and indicated mutants were examined during growth. Actin served as the loading control. **b** Quantification of Rad9 protein stability during normal growth. Rad9 protein levels examined during the time course as exemplified in panel A were quantified in reference to the loading control of actin levels. Averages and SEMs are shown based on at least two biological duplicates; statistical analysis for each time point was performed by one-tailed unpaired Student’s t-test. * p values <0.05. **c** Rad9 protein stability was examined when cells were treated with MMS and CHX. **d** Quantification of Rad9 protein stability in MMS-treated conditions. Experiments were done as panel A and data are presented as panel B except that cells were treated with MMS during the time course. Averages and SEMs are shown based on at least two biological duplicates; statistical analysis for each time point was determined by one-tailed unpaired Student’s *t*-test. * p values <0.05.

We next investigated the mechanisms by which the Mms22-Rtt107 interaction can promote DDC dampening. As Mms22 is a subunit of the Rtt101^Mms1^ ubiquitin E3^27^, a likely means for it to downregulate the checkpoint is through protein degradation. Given the strong genetic suppression of *mms22*^*RIM*^ by *rad9* and *ddc1* mutant alleles (Figs. 2a and 2c–2e, Supplementary Fig. 2), we examined the stability of these two proteins. We performed a standard procedure that utilizes cycloheximide (CHX) to block new protein synthesis, thus allowing the monitoring of protein stability during a time course. We first examined Ddc1 and Rad9 during normal growth. While the Ddc1 protein level showed minimal changes during an eight-hour time course, the Rad9 protein level exhibited a strong reduction over time (Figs. 4a and 4b, Supplementary Fig. 4a). Importantly, Rad9 instability is improved by the removal of Rtt101 or Rtt107, and by *mms22*^*RIM*^ (Fig. 4a). Similar improvement was also seen in *mms22Δ* and *mms1Δ* cells (Supplementary Fig. 4b). Rad9 protein level quantification based on at least two biological isolates per genotype showed that all examined mutants stabilized Rad9 levels to a similar degree (Fig. 4b and Supplementary Fig. 4c). In contrast to this group of mutants, Rad9 degradation was not affected by the lack of Slx4 (Supplementary Fig. 4d). We thus conclude that the Rtt107-Mms22-Rtt101^Mms1^ axis but not the Rtt107-Slx4 axis affects Rad9 protein stability. As mutants of Rtt107, Mms22, and Rtt101 did not fully stabilize Rad9, additional means also exist to promote Rad9 degradation.

We next monitored Rad9 stability when cells were treated by MMS. Again, we observed Rad9 degradation in wild-type cells and this was reduced upon the removal of Rtt107, Rtt101, and in *mms22*^*RIM*^ cells (Figs. 4c and 4d). This group of mutants also showed persistent Rad9 phosphorylation, which is catalyzed by the Mec1 kinase and serves as a marker for DDC activation (Fig. 4c)^21, 35^. These results are consistent with data presented above and further support the conclusion that the Mms22-Rtt107 interaction is required for degrading Rad9. Collectively, our findings suggest that the Mms22-Rtt107 interaction as well as the Rtt101^Mms1^ E3 are partly responsible for Rad9 degradation.

### Rad9 degradation is mediated by proteasomes

**Figure 5:**
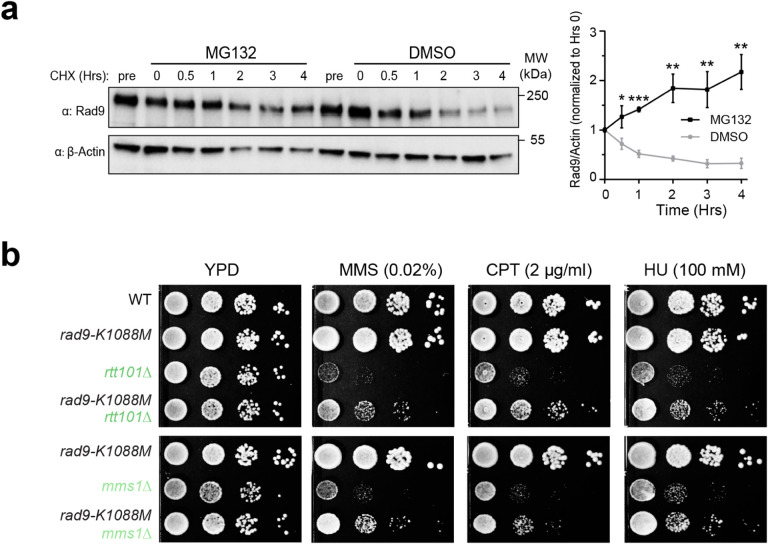
Rad9 degradation is proteasome-dependent and a *rad9* mutant rescues the MMS sensitivity of the Rtt101^MMS1^ E3 mutants. **a** Examination of Rad9 protein stability in the presence of MG132 that blocks proteasomal functions or DMSO control. Cells were simultaneously treated with cycloheximide (CHX) to block new protein synthesis. Left, a representative immunoblotting result showing that Rad9 was better stabilized in the presence of MG132 compared with the DMSO treatment. Right: quantification of Rad9 protein levels during the time courses in reference to the loading control of actin levels from three biological duplicates. Averages and SEMs are shown based on at least three biological duplicates; statistical analysis for each timepoint was performed by one-tailed unpaired Student’s *t*-test. * p <0.05, ** p< 0.01, *** p<0.001. **b** Sensitivity of *rtt101Δ* and *mms1Δ* cells toward three genotoxins is rescued by reducing Rad9 function via the *rad9-K1088M* mutation. Experiments were done as in Fig. 1b.

To further test the above notion, we asked whether Rad9 protein instability is mediated by the proteasomes. If Mms22 and Rtt107 collaborate with the Rtt101^Mms1^ E3 in Rad9 degradation, we would expect that blocking proteasomal functions can hinder Rad9 degradation. We treated cells with MG132 that inhibits proteasomal activity, and with CHX that blocks new protein synthesis. In the control DMSO treatment, Rad9 protein level reduced during a four-hour time course (Fig. 5a). However, the Rad9 protein was stabilized in the presence of MG132 (Fig. 5a). This result suggests that Rad9 degradation is mediated by proteasomes. This conclusion is in line with the involvement of the Rtt101^Mms1^ E3 in Rad9 degradation in cells.

#### rad9-K1088M rescues the MMS sensitivity caused by the loss of Rtt101 and Mms1

We next examined the functional significance of the Rtt101^Mms1^ E3’s involvement in Rad9 degradation. If this role is important for cell survival in genotoxins, we would expect that as seen for *mms22*^*RIM*^ mutant, genotoxin sensitivity caused by the loss of the Rtt101^Mms1^ E3 could be rescued by a mildly defective Rad9 allele. Indeed, we found that *rad9-K1088M* greatly increased the viability of either *rtt101Δ* or *mms1Δ* mutant cells on media containing MMS, CPT, or HU (Fig. 5b). This data provides evidence that like Mms22, Rtt101^Mms1^ E3’s involvement in Rad9 degradation can also be important for cellular survival in the face of genotoxins.

### Mms22-Rtt107 interaction helps the Rtt101^Mms1^ E3 associate with chromatin and regulating Rad9 stability on chromatin

Our data thus far suggest that Rtt107 dampens DNA damage checkpoint signaling through binding to Mms22, in addition to binding to Slx4. Previous studies have shown that Rtt107 helps to recruit both Mms22 and Slx4 to chromatin via its ability to recognize γH2A^14, 29, 36, 37^. These findings raise the possibility that Rtt107 binding to Mms22 may help the chromatin association of the Rtt101^Mms1^ E3 for degrading Rad9 on chromatin.

**Figure 6:**
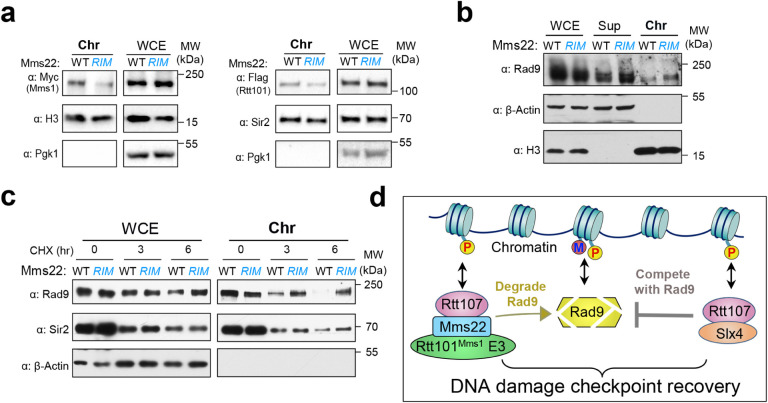
The Mms22-Rtt107 interaction promotes chromatin association of the Rtt101^MMS1^ E3 and degradation of chromatin associated Rad9. **a** *mms22*^*RIM*^ cells show increased level of Rad9 on chromatin. Histone H3 and β-Actin mark chromatin-bound and non-chromatin-bound protein samples, respectively. **b** Disrupting the Mms22 and Rtt107 binding via *mms22*^*RIM*^ reduces the chromatin association of Mms1 and Rtt101. Histone H3 or Sir2 marks chromatin-bound samples, while and Pgk1 marks non-chromatin-bound protein samples. **c** *mms22*^*RIM*^ leads to the stabilization of Rad9 on chromatin. Degradation of Rad9 in chromatin fraction and whole cell extract (WCE) was examined in the presence of CHX that blocks protein synthesis. Sir2 and Actin mark chromatin-bound and non-chromatin-bound protein samples, respectively. **d** A model suggesting that Rtt107 binding to nucleosome markers such as γH2A helps to recruit Mms22-containing Rtt101^Mms1^ ubiquitin E3 to chromatin that can facilitate Rad9 degradation. This mechanism acts in parallel with the Slx4-Rtt107 mediated displacement of Rad9 from chromatin. The combined effects of these two Rtt107-mediated pathways can provide more potent Rad9 silencing than either pathway alone in reducing the DNA damage checkpoint signaling. P: phosphorylation on γH2A; M: methylated H3K79

We tested the above notion first by examining the chromatin association of Rtt101 and Mms1 when the Mms22-Rtt107 interaction is disrupted by *mms22*^*RIM*^. We used a well-established chromatin fraction method to separate chromatin and soluble fractions^38^. We found that *mms22*^*RIM*^ not only reduced its own chromatin association but also lessened those for Rtt101 and Mms1 (Fig. 6a). We moved on to monitor Rad9 levels on chromatin and found that *mms22*^*RIM*^ cells exhibited an increased amount of Rad9 on chromatin (Fig. 6b), suggesting that the Mms22-Rtt107 interaction is required for Rad9 loss from chromatin. Importantly, in the presence of CHX that allows the examination of protein degradation, we found that degradation of Rad9 in the chromatin fraction was largely blocked in *mms22*^*RIM*^ cells (Fig. 6c). The Rad9 stabilization effect conferred by *mms22*^*RIM*^ is much stronger for the chromatin pools of Rad9 compared with the whole cell extract (WCE) pool of Rad9 (Fig. 6c), suggesting that the main effect of Mms22-Rtt107 stems from the regulation of the stability of Rad9 in the chromatin fraction. Collectively, these data provide evidence that the Mms22-Rtt107 interaction is important for the chromatin association of the Rtt101^Mms1^ E3 and degrading Rad9 in the chromatin fraction.

### Mms22-Rtt107 binding does not affect the Rad53-Asf1 or Rtt107-Dpb11 association

The Rtt101^Mms1^ E3 was previously shown to collaborate with Asf1 in down-regulating DDC when cells suffer from two double-strand DNA breaks (DSBs)^39^. The proposed model suggests that Rtt101^Mms1^ E3 may help Asf1 binding to Rad53, an interaction that could disfavor Rad53 phosphorylation thus leading to reduced DDC levels^39^. To discern if the Mms22 collaboration with Rtt101^Mms1^ during DDC dampening might be related to Asf1-Rad53 association, we examined this interaction by co-immunoprecipitation. We found that the amount of Asf1 recovered from Rad53 co-immunoprecipitation was similar between *mms22*^*RIM*^ and wild-type cells, in both normal growth and MMS treated conditions (Supplementary Fig. 5a). This result suggests that the effect of the Mms22-Rtt107 interaction on DDC dampening is not via regulating the Asf1-Rad53 interaction. Finally, considering that Rtt107 also associates with another checkpoint factor, Dpb11^23^, we asked whether this interaction is perturbed in *mms22*^*RIM*^ cells. We found that while *mms22-RIM* disrupted the Rtt107-Mms22 interaction, it did not affect the Rtt107-Dpb11 association in the presence or absence of MMS treatment (Supplementary Fig. 5b). These results suggest the DDC recovery role of the Rtt107-Mms22 interaction is not mediated by altering the Rtt107-Dpb11 interaction.

## DISCUSSION

Cellular survival of genotoxin treatments depends on the induction of the DNA damage checkpoint as well as its subsequent downregulation. While many studies have examined DDC induction, DDC downregulation is less understood. Here we describe a DDC dampening strategy that is mediated by the degradation of a central DDC mediator protein, Rad9, in yeast. We identified the players that contribute to this regulation, including the scaffold protein Rtt107, the Mms22 substrate adapter of the Rtt101^Mms1^ ubiquitin E3, and the ubiquitin E3 itself. Results from genetic suppression and checkpoint assays strongly support the conclusion that the Mms22-Rtt107 interaction provides an important means to downregulate DDC. Further, biochemical evidence suggests that Mms22 and Rtt107 collaborate with the Rtt101^Mms1^ E3 to facilitate proteasome-mediated degradation of Rad9. Interestingly, this regulation was more prominently seen for the chromatin pool of Rad9 compared with the non-chromatin pool. The preferential effect can be explained by our finding that the Mms22-Rtt107 interaction promotes the chromatin association of the Rtt101^Mms1^ E3. Based on these data, we propose a model wherein Rtt107 recruits the Mms22-containing Rtt101^Mms1^ E3 to chromatin to facilitate Rad9 degradation (Fig. 6d). Chromatin targeting of the Mms22-containing E3 could be achieved through previously identified bifurcated interactions of Rtt107, with its tetra-BRCT domain binding to Mms22 and additional binding sites recognizing γH2A and the phosphorylated H4T80 mark^29, 36, 40^. Finally, we provide evidence to support the notion that the Rtt107-Mms22-Rtt101^Mms1^ E3 axis acts in parallel with the Rtt107-Slx4 axis that competes with Rad9 for binding to nucleosomes. As such, Rtt107 could coordinate a two-pronged strategy that exploits distinct anti-Rad9 effects to downregulate DDC (Fig. 6d).

Rtt107 is required for chromatin targeting of both Mms22 and Slx4, which compete for the same binding site on the Rtt107 tetra-BRCT domain^29^. Despite the competition, both the Mms22-Rtt107 and Slx4-Rtt107 complexes are detected in cells^26^. Thus, Rtt107 is likely present in sufficient amounts to allow the formation of the two complexes. In line with this notion, the loss of Slx4-Rtt107 binding had no effect on the abundance of Mms22 on chromatin (Fig. 3d and Supplementary Fig. 3c). However, disrupting the Mms22-Rtt107 association increased Slx4 levels on chromatin (Fig. 3d and Supplementary Fig. 3c). These observations suggest a unidirectional modulation of the dynamics of the Rtt107 interactome that is worthwhile for further testing in the future. Most relevant here, despite showing increased chromatin association of Slx4, which could favor DDC downregulation, *mms22*^*RIM*^ cells nevertheless showed persistent checkpoint (Fig. 3d and Supplementary Fig. 3c). These data support our conclusion that the DDC dampening defect caused by *mms22* mutants is not due to lessening Slx4 chromatin association and vice versa. Rather, Mms22 and Slx4 each employ a different strategy to downregulate the checkpoint. While the Slx4-Rtt107 axis can disfavor Rad9 binding to chromatin^14, 23^, our result suggests that the Mms22-Rtt107 axis regulates Rad9 degradation. Interestingly, while *mms22*^*RIM*^ led to a general increase in Rad9 stability, it had a more prominent effect on the chromatin pool of Rad9, which is required for DDC signaling (Fig. 6c). This makes it possible that targeting this pool of Rad9 can make the Mms22-Rtt107 axis more efficient in downregulating the checkpoint. We note that we cannot exclude additional roles of Mms22-Rtt107 in affecting Rad9 association with chromatin. Nonetheless, since Slx4 did not affect Rad9 stability, regulation of Rad9 stability appears to be rather specific to the Mms22-Rtt107 axis and does not reflect a general feature of DDC dampening factors.

Using a Slx4 mutant specifically abolishing its binding to Rtt107, our data strengthened the previous conclusions regarding the role of Slx4 and Rtt107 in checkpoint dampening. Further, we show that the Slx4-Rtt107 interaction had a stronger effect when the Mms22-Rtt107 interaction was lost (Figs. 3a and 3b). In addition, *slx4*^*TTS*^ cells showed slower S phase progression compared with wild-type cells. This effect can be related to findings that Slx4 and Rtt107 associate with chromatin behind the replication fork to help replication in MMS conditions^37^. In contrast, *mms22*^*RIM*^ had no obvious defect in S phase progress but delayed exiting from the G2/M phase and increased levels of Rad53 activation (Figs. 3a and 3b). These observations provide additional support for the conclusion that Rtt107 can participate in two DDC regulatory pathways involving either Mms22 that mainly affects G2/M exit or involving Slx4 that has roles in controlling S phase progression.

The proteins examined here, including Rtt107, Mms22, Rtt101^Mms1^ and Slx4 are all multi-functional and each has several binding partners^25, 28, 30, 41^. In addition to our findings here, the Rtt101^Mms1^ E3 has been suggested to downregulate DDC via regulating the Asf1-Rad53 interaction^39^. Using the *mms22*^*RIM*^ separation-of-function allele, we showed that the Rtt107-Mms22 collaboration with Rtt101^Mms1^ E3 does not affect the Asf1-Rad53 association (Supplementary Fig. 5a). These data provide evidence that the Rtt101^Mms1^ E3 has at least two roles in checkpoint dampening, one through Rad9 degradation and the other via regulating the Asf1-Rad53 interaction. We also found that *mms22*^*RIM*^ did not affect the Rtt107 interaction with another checkpoint mediator Dbp11 known to also bind to Slx4 (Supplementary Fig. 5b). These data highlight the importance of using separation-of-function alleles in clarifying the role(s) of complex protein-protein interactions employed in DDC and genome maintenance.

Our data suggest that additional ubiquitin E3(s) can also help proteasome-mediated degradation of Rad9. It is common that one substrate is subjected to multiple E3 regulations to ensure their optimal degradation. Future studies aiming to identify additional factors involved in Rad9 degradation can expand our understanding of protein-level control of this key checkpoint mediator. Though our work focuses on the role of the Mms22-Rtt107 interaction in DNA damage conditions, we found that disruption of this interaction by *mms22*^*RIM*^ also affects genome maintenance during growth. In particular, *mms22*^*RIM*^ led to increased genomic instability at both rDNA and non-rDNA sites in the absence of genotoxin and showed additive effect with *slx4*^*TTS*^ (Supplementary Figs. 3a and 3b). This genetic finding corroborates the biochemical data that Rad9 stability is similarly regulated by Mms22 during growth and under genotoxin treatment, suggesting that Rad9 is a general client for the Mms22-Rtt101^Mms1^ E3 and its degradation is not triggered by DDC (Figs. 4a and 4b). Together, these data raise the possibility that the genome-protecting roles of the Rtt107-Mms22 axis during growth are also related to limiting Rad9 levels. It is currently unclear how limiting Rad9 levels during growth is beneficial to cells. Given that the Mec1 kinase shows local activation but does not trigger global DDC during normal S phase, we speculate that Rad9 degradation may help restrain Mec1 activation to a local level, thus preventing unnecessary and harmful full-on DDC^3, 42^. In this scenario, DDC dampening mechanisms may not only be critical for long-term survival in genomic stress conditions but could also help manage genome stability during growth. Future studies to explore these possibilities can provide a fuller picture to better unite DDC regulation in both conditions. Given the conservation of the protein factors examined here, results of our study can stimulate the studies of how higher eukaryotes can utilize protein degradation as a tool to downregulate checkpoint, and of the functional relevance of such regulation in tumorigenesis and during normal development.

## METHODS

### Yeast strains and genetic manipulation.

Yeast strains used in this work are listed in Table S1 and they are derivatives of W1588–4C, a RAD5 variety of W303 (*MATa ade2–1 can1–100 ura3–1 his3–11,15 leu2–3,112 trp1–1 rad5–535*)^43^. Protein tagging, gene deletion, and mutant alleles were generated at the endogenous genomic loci following standard PCR-based or CRISPR–Cas9 based methods^29^. All genetically altered loci were verified by sequencing. Standard yeast genetic manipulation was used for tetrad analyses and all experiments were conducted at 30°C unless noted. At least two different biological isolates per genotype were used for each assay.

### Genotoxin sensitivity tests.

Spotting assays to detect DNA damage sensitivity were carried out as described previously^44^. In brief, yeast cultures were grown overnight at 30°C in rich medium (YPD). Cultures were then diluted to OD_600_ 0.15 and allowed to grow to early mid-log phase in YPD. Subsequently, cells were spotted in 10-fold serial dilutions on plates containing YPD with or without DNA damaging agents at the indicated concentrations. Plates were incubated at 30°C and photographed after 2–4 days.

### Synchronization and cell cycle analysis.

The cell synchronization procedure was used as previously described with a few modifications^45^. In brief, early-mid log-phase yeast cultures grown in the YPD media at OD_600_ ~ 0.2 were treated with α factor for G1 arrest. After the initial addition of 5 μg/ml α factor (Thermo Fisher) for one hour, a second dose of 2.5 μg/ml α factor was added for another hour. Cell morphology was monitored to confirm G1 arrest. Subsequently, cells were released from G1 arrest by growing in YPD media containing 100 μg/ml protease (Sigma) to degrade α factor. The media also contained 0.03% MMS for genotoxin treatment. After 45 min, MMS was washed out, and cells were allowed to grow in YPD media. Samples were taken every 20 min for FACS analyses and Rad53 examination. FACS analyses were done as previously described^45^. Briefly, cells were fixed in 70% ethanol for 1 h at room temperature or overnight at 4°C. RNAs and proteins were degraded by sequential treatment with RNase A (Sigma) and Protease K (Sigma). DNA was stained with Sytox Green (Invitrogen). Samples were examined using a BD LSR II flow cytometer (BD). Data analyses were performed using the FlowJo Software (BD).

### Protein extraction to examine Rad53 phosphorylation.

Protein extracts were made using a TCA (Trichloroacetic acid) method to maintain Rad53 phosphorylation as described previously^46^. In brief, cells were homogenized using 0.5 mm silica beads (BioSpec Products) in the presence of 20% TCA on a FastPrep-24 bead-beating grinder (MP Biomedicals). After removing the supernatant by centrifugation, the precipitated proteins were washed with 5% cold TCA, and then dissolved in protein loading buffer (50 mM Tris-Cl pH 6.8, 2% SDS, 10% glycerol, 5% β-mercaptoethanol, 0.05% bromophenol blue). Samples were boiled for 5 min before being loaded onto 4–20% SDS-PAGE gels (Bio-Rad) and subsequently examined by immunoblotting (see below).

### Co-immunoprecipitation.

Standard protocols were followed to perform co-immunoprecipitation. In brief, cells were disrupted by bead beating in lysis buffer that contained 20 mM HEPES-KOH (pH 7.5), 100 mM KOAc, 1% Triton X-100, 2 mM Mg(OAc)_2_, 1 mM NaF, 2 mM β-glycerophosphate, and EDTA-free protease inhibitors (Roche). Lysates were cleaned by centrifugation at 20,000g for 30 min to obtain whole-cell extract, which was then incubated with anti-Flag beads (Sigma-Aldrich) or anti-HA beads (Pierce) for 2 h at 4°C. After washing the beads three times with lysis buffer, bead-bound proteins were eluted using loading buffer as described above. Proteins were boiled for 5 min before being loaded onto 4–20% gradient gels (Bio-Rad) for electrophoresis and immunoblotting (see below).

### Immunoblotting and antibodies used.

Proteins were transferred from gels to 0.2 μm nitrocellulose membranes (GE) before immunoblotting. The antibodies used include: anti-phosphorylated active Rad53 (F9, a gift from Marco Foiani and Daniele Piccini), anti-Ddc1 (a gift from Marco Muzi-Falconi), anti-Dpb11 (a gift from Dirk Remus), anti-Rad9 (a gift from John Petrini), anti-Histone H3 (ab1791, Abcam), anti-Actin (C4, 8691001, MP Biomedicals), anti-Sir2 (yN-19, sc-6666, Santa Cruz), anti-Flag (M2, F1804, Sigma), anti-HA (F-7, sc-7392, Santa Cruz), anti-Myc (9E10, BE0238, Bio X Cell), anti-TAP (PAP, P1291, Sigma), anti-Pgk1 (22C5, Molecular Probes), and anti-Tubulin (YL1/2, ab6160, Abcam),

### Chromatin fractionation.

Chromatin fractionation was performed as described previously with a few modifications^47^. In brief, yeast spheroplasts derived from log-phase cells were lysed using extraction buffer (150 mM KOAc, 20 mM pH 6.6 PIPES-KOH, 2 mM Mg(OAc)_2_, 1 mM NaF, 0.5 mM Na_3_VO_4_, 1% Triton X-100) supplemented with protease inhibitor cocktail (Sigma) by incubating on ice for 5 min in the presence Zymolyase. Lysates were then subjected to centrifugation at 16,000 g for 15 min at 4°C on a sucrose cushion. The resultant chromatin pellets were washed and resuspended with extraction buffer. Loading buffer was added to each sample before boiling for 5 min. Samples were then examined by SDS-PAGE followed by immunoblotting. Tubulin or Pgk1 was used as a marker of the non-chromatin fraction, while histone H3 or Sir2 was used as a marker for chromatin-associated proteins.

### Protein stability assay.

Protein stability was examined using a standard protocol as described previously^48, 49^. Briefly, yeast cultures were grown to OD_600_ 0.2 before CHX was added to a final concentration of 100 μg/ml to inhibit protein synthesis. Equal numbers of cells were collected every 1 h following CHX addition. Cell lysates were examined using the TCA method described above. The band intensity of each protein after immunoblotting was quantified using the FIJI software^50^.

### rDNA marker loss and GCR assays.

Standard protocols were followed for both assays. For the GCR assay that assesses the loss of the *URA3* and *CAN1* markers inserted at the YEL068c locus, at least six cultures were examined for each genotype^29, 51^. Cells were plated on SC (synthetic complete) media containing 5-FOA to counter-select *URA3* and containing canavanine to counter-select *CAN1*. The numbers of colonies grown on 5-FOA- containing SC (FC) plates reflect those that lost *URA3* and *CAN1* markers. Cells were also plated on SC plates to determine the total viable colonies. GCR rates were calculated as *m/N*_*T*_ by the following formula: m[1.24 + ln(m)] = N_FC_. Here, m represents mutational events, N_FC_ is the number of colonies on FC plates, and N_T_ is the number of colonies on SC plates. The upper and lower 95% confidence intervals were derived. Frequencies for the loss of the *ADE2* and *CAN1* markers inserted into the rDNA array were measured as described previously^52^. Cells were grown to stationary phase over equal doubling times and plated on SC media to count total viable colonies. Cells were also plated on media containing canavanine (SC + Can) to select those that lost the CAN1 marker. The frequency of marker loss was calculated as: F = N_can_/N_C_, where N_Can_ is the number of colonies on SC + Can plates and N_C_ is the number of cells plated on SC plates^53^.
